# An outbreak of norovirus gastroenteritis associated with a secondary water supply system in a factory in south China

**DOI:** 10.1186/1471-2458-13-283

**Published:** 2013-03-28

**Authors:** Yuan Li, Hongxiong Guo, Zhenghui Xu, Xiaotao Zhou, Hailong Zhang, Lijie Zhang, Jing Miao, Yi Pan

**Affiliations:** 1 Department of infectious disease prevention and control, Shenzhen bao’an center for disease control and prevention, 116 Longjinger Road, Shenzhen, 51810, China; 2 Department of STD and AIDS Prevention and Control, Jiangsu provincial center for disease control and prevention, 172 Jiangsu Road, Nanjing, 210009, China; 3 Department of comprehensive prevention and control, Shanhai Huangpu center for disease control and prevention, 181 Zhizaolu Road, Shanghai, 200011, China; 4 Department of epidemiology, Shenzhen bao’an center for disease control and prevention, 116 Longjinger Road, Shenzhen, 518100, China; 5 Microbiology laboratory, Shenzhen center for disease control and prevention, 8 Longyuan Road, Shenzhen, 518055, China; 6 The office of Chinese field epidemiology training, Chinese center for disease control and prevention, 155 Changbai Road, Beijing, 102206, China; 7 The institute of virology, Chinese center for disease control and prevention, 155 Changbai Road, Beijing, 102206, China; 8 Rollins School of Public Health, Emory University, 1518 Clifton Road, Atlanta, 30322, USA

**Keywords:** Norovirus, Acute gastroenteritis, Outbreak, Secondary water supply system

## Abstract

**Background:**

Between September 17 and October 3, 2009, hundreds of workers employed in a manufacturing factory in Shenzhen, a city in south China developed a sudden onset of acute gastroenteritis. A retrospective cohort study is designed to identify the risk factors and control this outbreak.

**Methods:**

Information on demographic characteristics, working place, the history of contact with a person having diarrhea and/or vomiting, drink water preference and frequency, eating in the company cafeteria or outside the company, hand-washing habits and eating habits is included. Furthermore, in order to find the contamination source, we investigated the environment around the underground reservoir and collected water samples from the junction between municipal supply water system and underground reservoir to test potential bacteria and virus, examine the seepage tracks on the wall of the underground reservoir from the side of septic tank, and check the integrity and attitude of this lid. Relative risk was presented and Chi-square test was performed. All the analyses were performed with OpenEpi software version 2.3.1 online.

**Results:**

The cohort study demonstrated that the workers who had direct drink water were 3.0 fold more likely to suffer from acute gastroenteritis than those who consumed commercial bottled water. The direct drinking water, water of the tank of buildings, and the underground reservoir were positive only for norovirus. Norovirus was also detected from stool and rectal swab samples from patients with acute gastroenteritis. The underground reservoir was found to be the primary contamination source. Further environmental investigation showed that the norovirus contaminated substance entered into the underground reservoir via access holes in lid covering this underground reservoir.

**Conclusion:**

This acute gastroenteritis outbreak was caused by the secondary supply system contaminated by norovirus in this factory. The outbreak of gastroenteritis cases caused by norovirus frequently occurred in China due to a lack of surveillance and supervision, and due to faults in the construction of such water systems. Therefore, more attentions should pay to the secondary supply water system in China.

## Background

Norovirus is a dominating cause of gastroenteritis in adults and older children. Characteristic symptoms of norovirus consist of nausea, vomiting, and diarrhea generally appear after an incubation period of 24–48 hours and last for about 48–72 hours. Outbreak of gastroenteritis caused by norovirus infection was first discovered in 1968, and had been reported in many places around the world [1-3]. Norovirus also resulted in 50% of all food-borne outbreaks of gastroenteritis in the USA [4]. In addition, norovirus is highly contagious as a few virions are sufficient to cause an illness [5]. Direct contact with vomitus or feces of infected persons, sharing food, water, and/or utensils, and contact with a contaminated environment are all possible routes of norovirus transmission [6-8]. However, outbreaks involving a large number of individuals are usually transmitted through common sources such as food and water [9,10].

Shenzhen is a city in Guangdong province, southern China and is next to Hong Kong. As the manufacturing boom, millions of migrant workers are employed in the manufacturing industry in Shenzhen. From September 17 2009, hundreds of workers in a manufacturing plant in Shenzhen City developed a sudden onset of vomiting and diarrhea. In order to control this outbreak, Shenzhen Bao’an Center for Disease Control and Prevention were invited to identify the causes of this outbreak. On September 26, we conducted a retrospective cohort study to investigate the factors associated with this acute gastroenteritis outbreak in order to identify the source of contamination and the infection route, and finally control and prevent such outbreak in the future.

## Methods

### Epidemiologic investigation

In this outbreak, study population consisted of suspected cases, probable cases and confirmed cases with the following definitions. Suspected cases were defined as company staff with an onset of vomiting or diarrhea (≥once/day) between September 14 and September 25, 2009. Probable cases were defined as those with an onset of diarrhea (≥three times/day) or vomiting (≥twice/day). Confirmed cases were those probable cases that were tested positive for norovirus by reverse transcription-polymerase chain reaction (RT-PCR). In this investigation, we defined diarrhea as having more than one loose or liquid stools per day, or as having more stools than normal for that person. A retrospective cohort study was conducted and possible risk factors were drinking water or food from the cafeteria in this factory. Information on drinking-water preference and frequency, eating in the factory cafeteria or not, hand-washing habits, eating habits, gender, age, workplace, history of contact with a person with diarrhea and/or vomiting were collected through a questionnaire. Drinking-water preference contained the following two categories: drinking water from directly drinkable-water dispensers (DDWDs) or commercial bottled water. This study was approved by the Ethics Committee of Shenzhen Bao’an Center for Disease Control and Prevention (2009010).

### Laboratory tests

Samples from 29 water specimens were collected, including 13 from the water tanks on the top of five buildings, nine from drinking-water machines, five from bottled water, and two from the underground reservoir. All samples were transported to Bao’an District Center for Disease Control and Prevention. All water samples were tested according to the Standards for Drinking Water Quality (GB5749-2006), including the detection of total coliforms, thermotolerant coliforms, *Escherichia coli*, *Salmonella, Shigella*, *Campylobacter*, and *Yersinia enterocolitica*[11]. In addition, we also tested for norovirus and rotavirus.

As we suspected noroviruses were the responsible agent, we began to collect the specimens of patients. A total of 12 stool and 9 rectal swab samples were collected to test for norovirus. These samples were also transported into Bao’an District Center for Disease Control and Prevention, and detected for norvirus and rotavirus.

### RNA extraction and amplification

Virus concentrations of water samples were based on positively-charged filters from 1-l samples, as described previously [12]. Approximately 50–80-μg stool samples were weighed, diluted 1:10 in nuclease-free H_2_O, and then vortexed for 30 s. Samples were clarified by centrifugation at 6,800 × *g* for 10 min at room temperature. Viral RNA was extracted from 140-μl processed samples using a QIAamp Viral RNA kit (Qiagen, Victoria, Australia), according to the manufacturer’s instructions.

### Real-time fluorescence RT-PCR

Real-time fluorescence PCR for rotavirus and norovirus was performed using an ABI 7500 real-time PCR system with a commercial kit according to the manufacturer’s instructions (catalog numbers SA-6261 for rotavirus and SA-6251 for norovirus, Beijing Suoao Biotechnology Company Limited, Beijing, China).

### Environmental investigation and exclusion of pollution sources

The factory comprised an area of 350,000 m^2^, consisting of three buildings (A1, A2, A3) used as workshops, three buildings (A21, A22, A23) used as dormitories for workers, one building (A24) as a canteen, one building (A15) as a kitchen, and one building as a repair room. About 13,000 workers ate breakfast, more than 6,000 ate lunch, and over 11,000 ate dinner at the company cafeteria every day. Water was supplied from the municipal water supply system and stored in an underground reservoir with a capacity of 8,000 tons. Water from the underground reservoir was pumped into water tanks on top of eight buildings (A11, A1, A12, A3, A21, A22, A23, and A15), and then supplied to DDWDs provided at multiple sites in these eight buildings. Each floor of the dormitory buildings had two DDWDs.

**Figure 1:**
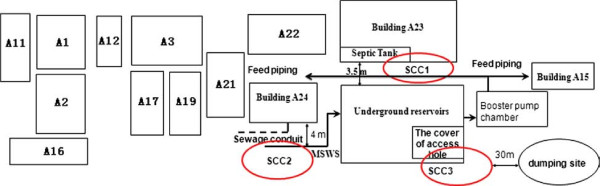
**Schematic of environment around underground reservoirs in the factory having gastroenteritis outbreak.** SCC1: the first suspected source of contamination. SCC2: the second suspected source of contamination. SCC3: the third suspected source of contamination. MSWS: Municipal supply water system. Building A1, A3, A11, A12, A15, A21, A22, A23 provide DDWD. Building A2, A16, A17, A19 provide bottled water.

The environment around the underground reservoir is shown in Figure 1. Three possible contamination sources are indicated by red circles. The sewer pipe was close to the municipal water supply pipe. To exclude contamination of the municipal supply water system from the sewer pipe, we collected water specimens from the junction between the municipal water supply system and the underground reservoir to test for bacteria and viruses. Secondly, to exclude contamination from the septic tank near building A23, we looked for seepage tracks from the side of the septic tank on the wall of the underground reservoir. Finally, we checked the integrity of the reservoir lid in order to determine whether contaminants had entered the underground reservoir through this lid.

### Statistical analysis

The distribution of major symptom in workers was summarized by frequency and percent. A retrospective cohort study was conducted to investigate possible risk factors for acute gastroenteritis among a workshop of employees in the company with outbreak of acute gastroenteritis. Chi-square test were performed using OpenEpi software version 2.3.1 online (http://www.openepi.com/OE2.3/Menu/OpenEpiMenu.htm). Relative risks (RR) and 95% confidence intervals (CI) were calculated.

## Results

### Descriptive epidemiology

A total of 396 workers developed gastrointestinal symptoms between September 17 and September 25, 2009. Diarrhea (77%) was the most common symptom (Table 1), and more than 30% of cases had abdominal distention, abdominal pain, or vomiting. But only few patients consulted a physician at the hospital. We collected 12 stool samples and nine rectal swab samples from 21 of these patients, of which 75% (9 of 12) of stool samples and 55.6% of rectal swab samples were positive for norovirus. None of the samples were positive for rotavirus.

**Table 1:** The distribution of major symptom in workers from September 14 through 25, 2009

**Symptom**	**n**	**%**
Diarrhea	308	77
Abdominal distention	156	39
Abdominal pain	154	39
Vomiting	140	35
Hypodynamia	104	26
Dizziness	50	13
Fever	22	5.5

**Figure 2:**
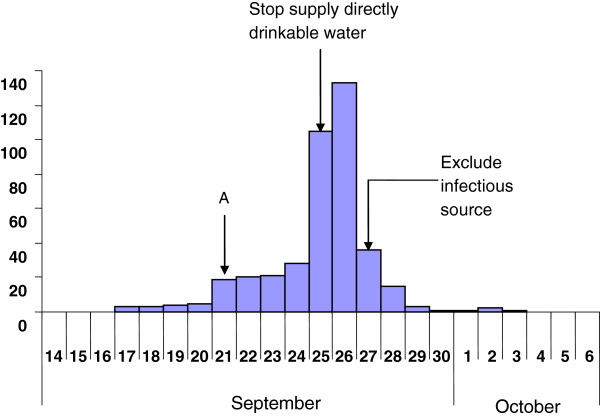
The time distribution of diarrhea cases amongst workers from September 17 to October 3, 2009.

The first case of gastroenteritis occurred on September 17, followed by a rapid increase in the number of cases over the following 4 days (Figure 2). In this factory, five buildings supplied DDWD, while four supplied commercially-bottled water. The incidence of gastroenteritis in the buildings that used DDWD was 2.9% (354/12388), compared to 0.8% (15/1999) in buildings that only provided bottled water (p < 0.0001).

### Risk factors

For the analysis of risk factors in the cohort study, we randomly selected a workshop of 380 employees. Of 380 employees, 38 rotated their days off; 27 declined to join this investigation. Finally 315 employees were interviewed. Of the 162 employees who used a DDWD, 41 (25%) developed gastroenteritis (RR: 3.0, 95% CI:1.7–5.3) (Table 2). In contrast, only one of six employees (17%) who drank bottled water developed gastroenteritis (RR:0.97, 95% CI: 0.16-5.9). The incidence of gastroenteritis among those who had contact with patients having gastroenteritis was the 6.1-fold (95% CI: 3.4–11) higher than those with no contact with infected persons. However, the number of non-responses to this question was high. We further investigated the reason for the lack of response to this question, and found it was difficult for those employees to report whether or not they had contact with vomit or feces from employees with gastroenteritis.

**Table 2:** Univariate analysis of risk factors for acute gastroenteritis among employees of a company in Shenzhen City, Sep. 14 to Oct. 3 2009

	**Non-Response**		**Numbers of subjects responding**				**Attack rates**			
	**%(n)**	**%(n)**	**Exposed**		**Not exposed**		**Exposed**		**Risk ratio**	**95% CI**^**#**^
	**Cases**	**Total**	**Cases**	**Total**	**Cases**	**Total**	**Yes**	**No**			
Drink										
DDWD^*^	0	0	41	162	13	153	25	8.5	3.0	1.7 ~ 5.3
Bottled water	0	0	1	6	53	309	17	1.7	0.97	0.16 ~ 5.9
Repast time in company										
Breakfast	0.0(1)	0.0(10)	48	274	6	41	18	15	1.2	0.55 ~ 2.6
Lunch	0	0.0(2)	53	311	1	4	17	25	0.68	0.12 ~ 3.8
Supper	0	0.0(5)	52	306	2	9	17	22	0.77	0.22 ~ 2.7
Food taken late at night	0.3(17)	0.3(88)	47	278	7	37	17	19	0.89	0.44 ~ 1.8
Contact with patients	86(25)	49(104)	12	22	17	189	55	9.0	6.1	3.4 ~ 11
Eating raw and cold food	0	0	13	47	38	236	28	16	1.7	1.0 ~ 3.0

^*^: DDWD is drinking direct water disposal.

#: 95%CI is 95% certificated interval.

### Laboratory tests

Of 29 water samples, 14 were positive for total coliforms. However, all water samples were negative for *E. coli, Salmonella, Shigella, Campylobacter, Y. enterocolitica*, and rotavirus. Twelve of 29 water samples were positive for norovirus, including four of six DDWD samples, two of three water samples from the underground reservoir, and four of five water samples from the tanks on top of the buildings. All five bottled water samples were negative for norovirus, rotavirus, *E. coli*, *Salmonella*, *Shigella*, *Campylobacter*, *Y. enterocolitica*, and total coliforms. All positive samples were collected from buildings A12, A13, A14, which only provided water via DDWDs.

After confirming norovirus as the organism responsible for the outbreak, we collected stool and rectal swab samples from patients with acute gastroenteritis and examined them for norovirus. Of 10 stool samples, five were positive for norovirus, and four of nine were positive for norovirus in rectal swab samples. All stool and rectal swab samples were negative for rotavirus.

Total coliforms were observed in 8 of 24 water samples, excluding five bottled water samples, and exceeded 100 colony-forming units/ml, which is the upper limit allowed by the Standard of Water Quality of China. All other indicators met the standard criteria.

### Environmental investigation

The distribution of the buildings around the underground reservoir is shown in Figure 1. The three potential contamination routes included the septic tank, seepage conduits, and the lid of the underground reservoir. There was no trace of seepage on the wall of the underground reservoir near to the septic tank, and we therefore excluded the septic tank as a possible source of contamination. Secondly, we found no defects in the conduit pipe so that it was excluded as a potential source. In addition, we checked the integrity of the reservoir lid, and noted eight access holes. Furthermore, the lid was at a similar height to the ground around the underground reservoir (Additional file 1), and there was no shed to protect the water from contaminants falling or flowing into the underground reservoir through these access holes; particles smaller than the access holes in the lid were therefore able to enter the water in the underground reservoir.

### Control measures

The supply of DDWD was stopped on September 25 2009, and the incidence of gastroenteritis decreased rapidly 2 days later (Figure 2). On September 27 2009, we suspected that the underground reservoir water was contaminated, and the supply of water from this reservoir was therefore stopped immediately. The occurrence of new cases then dropped rapidly within the next 3 days. Although three new cases still appeared between October 1 and October 2, these cases may have been infected with norovirus before September 29, given that the incubation period of norovirus can be 48–72 hours.

## Discussion

The results of this epidemiologic investigation indicated that the outbreak of acute gastroenteritis in the factory under investigation was caused by contamination of a secondary water supply system by norovirus. Noroviruses and total coliforms were detected in the underground reservoir water and the water tanks on top of the buildings, which in turn supplied the DDWDs. Employees in these buildings experienced significantly higher rates of acute gastroenteritis than those living in buildings that supplied bottled water.

To confirm the source of the contaminants in the underground reservoir, we investigated the surrounding environment and found eight access holes in the lid covering the reservoir. Furthermore, the lid was at a similar level to the ground around it, making it possible for contaminated substances to enter the underground reservoir through these access holes. Following the outbreak, a 1.2-m high cover has been erected over the reservoir to prevent contamination via the access holes. No new waterborne acute gastroenteritis cases were reported in this factory within the subsequent 2.5 years.

Although total coliforms in the secondary water supply system were above the upper limit required according to drinking water standards [11], no pathogenic bacteria were detected. Norovirus is a robust, chlorine-resistant virus, and 20 ppm chlorine causes no significant reduction in human norovirus infectivity [5,13,14]. Moreover, norovirus in groundwater remains infectious even after storage at room temperature in the dark for 61 days [15]. Meanwhile, norovirus has a high prevalence in groundwater and surface water [16,17]. Overall, these features of norovirus may explain why it is a more common causative agent of waterborne outbreaks of acute gastroenteritis than other viruses. Norovirus is the predominant cause of viral, waterborne outbreaks of acute gastroenteritis, especially in viral drinking-water outbreaks [18]. Secondary water supply systems are common in China. Although many surveillance systems are in place to detect indicator microorganisms in secondary water supply systems every 3 months, surveillance does not cover all such systems, and the detection interval is too long. Sporadic cases and outbreaks of acute gastroenteritis caused by norovirus contamination of drinking water are common in China [19-22], which suggests that norovirus contamination of secondary water supply systems may play an important role in the occurrence of these outbreaks.

There was a notable limitation of the current study. Contact with patients represents one of the major transmission routes for norovirus, but the number of non-responses to this question was high. We were therefore unable to fully investigate the role of transmission by contact in this outbreak.

To our knowledge, this study represents the first report of an outbreak of acute gastroenteritis caused by norovirus contamination of a secondary water supply system in China. Such water systems are common in China, and cases of norovirus-associated acute gastroenteritis are also frequent. However, the contamination sources responsible for most of these norovirus gastroenteritis cases have remained unidentified. The current results highlight the risk of contamination of secondary water supplies by robust norovirus.

## Conclusion

Secondary water supply systems in China represent potential sources of acute gastroenteritis outbreaks due to a lack of surveillance and supervision, and due to faults in the construction of such water systems. More attention should be paid to the design and supervision of secondary water supply systems to reduce the incidence of norovirus-related waterborne diseases.

## Abbreviations

DDWD: Directly drinkable water dispensers

## Competing interests

The authors declare that they have no competing interest.

## Authors’ contributions

YL design questionnaire, collected the data, conducted the main analyses, and participated in the writing of the manuscript. HG review and interpret the result of analyses, and edit the manuscript. ZX and HZ participated in analyses of data. XZ participated in collecting data. MJ and LZ contributed to design questionnaire and interpretation of analyses. YP re-edited this manuscript in English writing. All authors read and approved the final manuscript.

## Pre-publication history

The pre-publication history for this paper can be accessed here:

http://www.biomedcentral.com/1471-2458/13/283/prepub

## Supplementary Material

Additional file 1 The environment of underground reservoir and access holes of lid covering reservoir. Click here for file
