# Supplementary material for: An outbreak of norovirus gastroenteritis associated with a secondary water supply system in a factory in south China
Source: BMC Public Health. 2013 Mar 28;13:283. doi: 10.1186/1471-2458-13-283 (PMC3679847; doi:10.1186/1471-2458-13-283)
Supplement: Additional file 1 — The environment of underground reservoir and access holes of lid covering reservoir. [file 1471-2458-13-283-S1.doc]

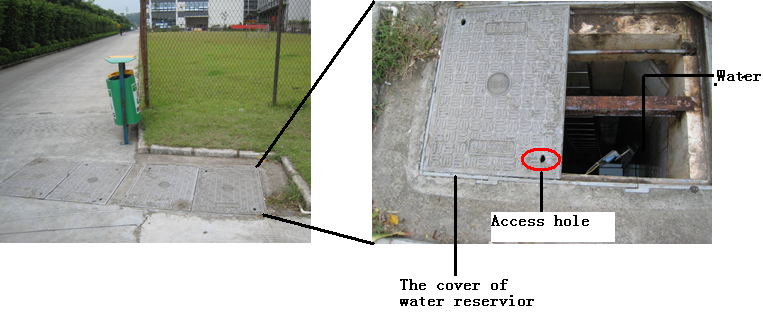


Supplement 1. The environment of underground reservoir and access holes of lid covering reservoir
